# Evidence of accelerated ageing in clinical drug addiction from immune, hepatic and metabolic biomarkers

**DOI:** 10.1186/1742-4933-4-6

**Published:** 2007-09-24

**Authors:** Albert Stuart Reece

**Affiliations:** 1 Southcity Family Medical Centre, 39 Gladstone Rd., Highgate Hill, Brisbane, Queensland, 4101, Australia; 2 Herston Medical School, Herston Rd, Herston, University of Queensland, Brisbane, Queensland, Australia

## Abstract

**Background:**

Drug addiction is associated with significant disease and death, but its impact on the ageing process has not been considered. The recent demonstration that many of the items available in routine clinical pathology have applicability as biomarkers of the ageing process implies that routine clinical laboratory parameters would be useful as an initial investigation of this possibility.

**Methods:**

12,093 clinical laboratory results 1995–2006 were reviewed. To make the age ranges of the medical and addicted groups comparable the age range was restricted to 15–45 years.

**Results:**

739 drug addicted (DA) and 5834 general medical (GM) age matched blood samples were compared. Significant elevation of immune parameters was noted in the C-reactive protein, erythrocyte sedimentation rate, total lymphocyte count, serum globulins and the globulin:albumin ratio (P < 0.01). Alanine aminotranferase, creatinine, urea, and insulin like growth factor-1 were also significantly higher (P < 0.01) in the DA group. Albumin, body mass index and dihydroepiandrosterone sulphate were unchanged and cholesterol was lower (all P < 0.05).

**Conclusion:**

These data demonstrate for the first time that addiction is associated with an altered profile of common biomarkers of ageing raising the possibility that the ageing process may be altered in this group. Infective and immune processes may be centrally involved. They suggest that addiction forms an interesting model to further examine the contribution of immune suppression and hyperstimulation to the ageing process.

## Background

Addictive disorders are well known to be associated with a high level of physical and psychological disorders [1]. Most chronic chemical addictions are also associated with very elevated rates of mortality [2] which have been estimated to be 10–70 [3,4] times that of non-clinical populations. Addictive drugs are known to impair cell growth and division [5-7] and therefore might be presumed to impact particularly dividing cells such as stem and progenitor cell pools [8,9]. They are also known either singly or in combination to potentiate apoptosis [10-14] thereby adding to this effect. Ageing medicine has recently emerged as a distinct scientific discipline. The cellular hypothesis of ageing suggests that the ageing phenotype of the organism is associated with cellular correlates of age related change including cell loss, reduced rates of cell renewal, and higher numbers of senescent, marginally functional, and non-replicative cells in the tissues [15,16]. Therefore if the growth inhibitory effects of addiction occur throughout the organism, one might reasonably expect that signs of accelerated ageing may be evident. Such a putative progeroid effect might be expected to underlie the elevated rates of morbidity and mortality observed clinically amongst addicts in much the same way as they do in geriatric populations. In this connection it is relevant that a variety of changes consistent with such a pro-ageing effect [17] have been reported both from our own clinic and elsewhere, and relating to neuropsychiatric disorders [18-20], calcific [21] and degenerative[22] arteriosclerosis, osteoporosis[23,24], oligospermia [25,26], hair greying [27] and severe dental and periodontal pathology [28,29]. Consideration of all of the clinical expressions of this generic hypothesis of the toxicology of addiction is beyond the scope of the present investigation.

The immune system in particular is central to a consideration of ageing. The immune system of drug dependent individuals is known to be suppressed, dysfunctional and hyperstimulated [30-34], and these perturbations are the subject of on-going enquiry. Not only does the immune system itself demonstrate age related change [35,36], but the ready access of its cells and cytokines to most parts of the body including the CNS in many inflammatory disease states, suggests that dysfunction in this system might potentially be an important mediator and contributor to putative age related processes including atherosclerotic [37] and neurodegenerative [38] disorders as well as a major source of free radicals [39]. Some chemical addictions have also been associated with free radical generation [40,41].

The potential implications of an improved understanding of the pathophysiology of addiction may have several clinically relevant implications. There is intense interest from the National Institutes of Ageing and others in the development of anti-ageing medicines (such as resveratrol and diaformin) [42] and it may be that such agents come to have a role in the treatment of the addictions. Conversely research in anti-ageing medicines has fuelled vigorous interest in various biomarkers as surrogate end points for otherwise lengthy clinical trials of putative agents [43]. Conveniently many of the common biomarkers are readily available in clinical pathology laboratory parameters in widespread use. If the classical chemical addictions are shown to accelerate ageing, then clearly there may be a role for their antagonists (such as the opioid antagonists naltrexone and nalmefene and cannabinoid antagonists such as rimonabant) which are largely pro-mitotic, as anti-ageing therapies if a deleterious effect on cancer can be avoided. And clearly a better understanding of the mechanism by which the various chemical addictions act to induce the aged phenotype may enhance our understanding of the basic pathophysiology of the ageing process itself, including gametotoxic and developmental implications.

My clinic sees both medical patients and patients addicted (mainly to opiates) and so provides an opportunity to compare relevant states of health and disease in these two populations. The present paper is presented as an initial clinical study of this subject and is intended to stimulate further investigative interest in the possible progeroid effects of addiction based on readily available clinical information relating to infective episodes and laboratory pathology.

## Methods

### Patient Recruitment, Survey and Sampling

This primary care medical centre operates in inner city Brisbane and sees both general medical (GM) and drug addicted (DA) patients. Most of our work involves the treatment of opiate addicted persons, and this is undertaken predominantly with the buprenorphine/naloxone combination sublingual tablet. Blood samples were taken opportunistically on patients as part of their routine clinical care.

### Laboratory Analysis

Specific haematological and biochemical data of interest was requested from our commercial clinical pathology laboratories for our patients in the period 1995–2006. Assays were done by standard clinical laboratory methods. The clinical laboratories used were Queensland Medical Laboratories (QML) and Mater Hospitals (MAH) Pathology Laboratories both in Brisbane, Queensland, Australia. Both laboratories are accredited within Australia by the National Association of Testing Authorities (NATA) to the current Australian medical laboratory standards (AS-15189), and QML is accredited to ISO 9001 the international clinical laboratory standard. 96% of the work was done at QML, and 4% at MAH. GM and DA samples were sent equally to both laboratories. All pathology samples were sent to QML prior to June 2006, and to MAH after that time. Where available results from both sites were combined unless the normal range or distribution of the results were significantly different so that artefactual differences were introduced into the analysis. In such cases, only the larger dataset was used. When data from the two laboratories was combined, care was taken not to alter the pattern of results. Reported data for albumin were based only on QML data. In other cases data from the two laboratories was directly combined. The normal ranges for the two laboratories are included in Table 1. Data for DHEAS and IGF1 are not included as they are age and sex dependent and vary substantially over the lifespan. The normal ranges for these two parameters in the two laboratories were thought to be similar.

**Table 1:** Normal ranges of clinical laboratory parameters

PARAMETER	QML Range	MAH Range
CRP (IU/l)	1–6	1–6
ESR (mm/hr)	1–25	1–25
Albumin (g/l)	35–50	33–47
Globulins (g/l)	20–40	20–40
Lymphocytes (*10^9^/l)	1.1–4.0	1.0–4.8
ALT (U/l)	0–45	5–45
Cholesterol (mmol/l)	3.1–6.5	2.6–5.5
Urea (mmol/l)	2.0–7.0	3.0–8.0
Creatinine (mcmol/l)	40–110	70–120

### Data Analysis and Statistics

Data was entered into Excel spreadsheets. Data is listed as mean ± S.D. or S.E. as indicated. Statistical analysis was performed using the EpiInfo program obtained from Centres for Disease Control for Chi squared comparisons of paired categorical data to calculate relative risks, 95% confidence intervals and significance levels; quantitative data was analyzed using T-tests performed in Microsoft Excel; and graphs were presented using the statistical software package "Statistica" on a Dell personal computer. Clinical pathology results were made available in electronic format. DA were sorted from GM patients by the presence of a Hepatitis C test, a test which is limited almost completely to use in DA patients. No data (such as outliers) was excluded from the analysis.

### Ethical Approval

All procedures and studies were consistent with good clinical practice as defined by the National Health and Medical Research Council and the Declaration of Helsinki and were approved by the approved by the Human Research Ethics Committee of the Southcity Family Medical Centre. Patients gave informed consent to participation in the study. No external source of funding was involved.

## Results

The age and sex distribution of the two groups is given in Table 2. In this study 798 DA and 11295 GM blood samples from patients of all ages were available for analysis. However, as indicated in Table 2 again problems of age disparity between the two groups were encountered. Therefore the age range was restricted to 15–45 years, leaving 739 and 5834 samples for consideration. The resulting mean ages (± S.E.) were 30.92 ± 0.24 and 30.74 ± 0.11 years respectively (P – N.S.).

**Table 2:** Demographic data

PARAMETER	ADDICTS	MEDICAL	(TOTAL/) SIGNIFICANCE
All Sample Size (Total)	798	11295	12093
Age as Mean(SD)	31.95 (8.39)	46.66 (19.18)	<0.0001
			
Sample Size <45 Years (Total)	739	5834	6573
Age	30.92 (6.52)	30.74 (8.63)	N.S.
% Male	73.5%	54.3%	<0.0001

Data as mean ± S.D.

The present data was not linked to a detailed drug use history. DA patients presented for management of opiate addiction, where the primary substance of abuse was 86% heroin, 12% morphine, 1% buprenorphine and 1% methadone. More detailed information relating to drug use by this cohort has been previously published and is included for convenience in Table 3. Only 1% of each group was HIV positive.

**Table 3:** Drug use

PARAMETER	ADDICTS	MEDICAL
Dose & Duration		
Cigarette Numbers	16.08 (10.87)	4.36 (8.12)***
Cigarette Duration (Yrs)	13.83 (8.62)	3.77 (8.145)***
Alcohol Size (g/d)	8.65 (21.51)	1.77 (10.23)***
Alcohol Duration (Yrs)	2.97 (5.84)	0.96 (3.08)***
Cannabis Size (g/d)	2.71 (5.25)	0.34 (1.56)***
Cannabis Duration (Yrs)	7.27 (7.57)	0.81 (3.24)***
Heroin Size (g/d)	0.57 (1.01)	0.00***
Heroin Duration (Yrs)	8.97 (6.92)	0.00***
Morphine Size (g/d)	0.05 (0.20)	0.00***
Morphine Duration (Yrs)	1.19 (4.12)	0.00***
Methadone Max. Dose	27.53 (47.19)	0.00***
Methadone Duration (Yrs)	1.21 (3.05)	0.00***
Amphetamine Size (g/d)	0.55 (1.51)	0.02 (0.15)***
Amphetamine Duration (Yrs)	1.89 (4.29)	0.19 (1.31)***
Dose-Duration Products		
Cigarette Index (Cig-Yrs)	256.25 (217.70)	69.26 (164.68)****
Alcohol Index (g-Yrs)	73.49 (219.19)	21.89 (132.79)*
THC Index (g-Yrs)	28.99 (62.87)	3.31 (14.53)****
Heroin Index (g-Yrs)	5.92 (11.06)	0****
Morphine Index (g-Yrs)	0.39 (1.57)	0***
Methadone Index (mg-Yrs)	114.26 (373.82)	0****
Amphetamine Index (g-Yrs)	1.39 (10.17)	0.19 (1.31)****

Data as Mean (± S.D.)

* – P < 0.05

*** – P < 0.001

**** – P < 0.0001

A study of this nature is predicated on being able to reliably separate intravenous drug using (IVDU) DA from GM. Review of 100 samples from each group found a 1% and 3% error rate respectively in group assignment based on HCV status. Furthermore the HCV positivity rate in the DA group was 69%, which accords well with 70% as previously found in studying DA in our clinic, suggesting minimal contamination and dilution of the IVDU DA group.

**Figure 1:**
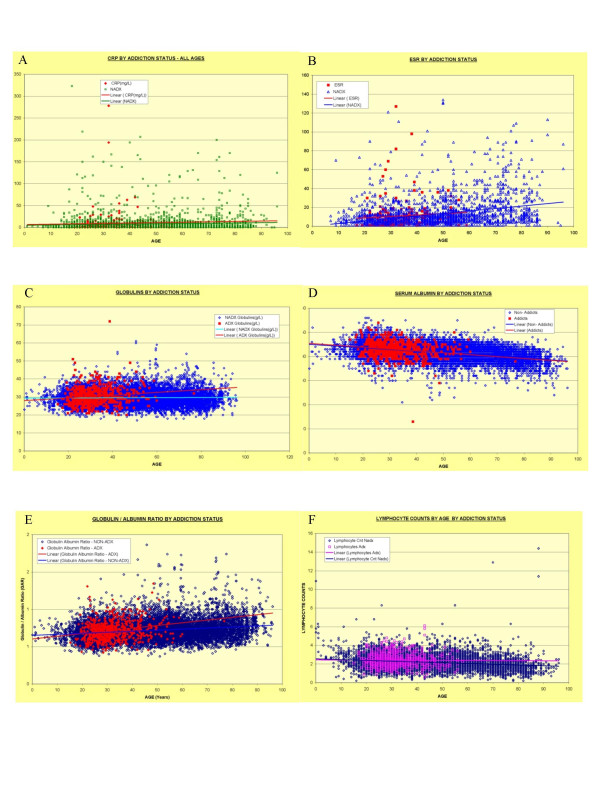
Laboratory Parameters of Addiction, All Ages: Part I – Immunity. A: C-reactive Protein – CRP; B: Erythrocyte Sedimentation Rate – ESR; C: Globulins; D: Albumin; E: Albumin Globulin Ratio; F: Lymphocyte Count

**Figure 2:**
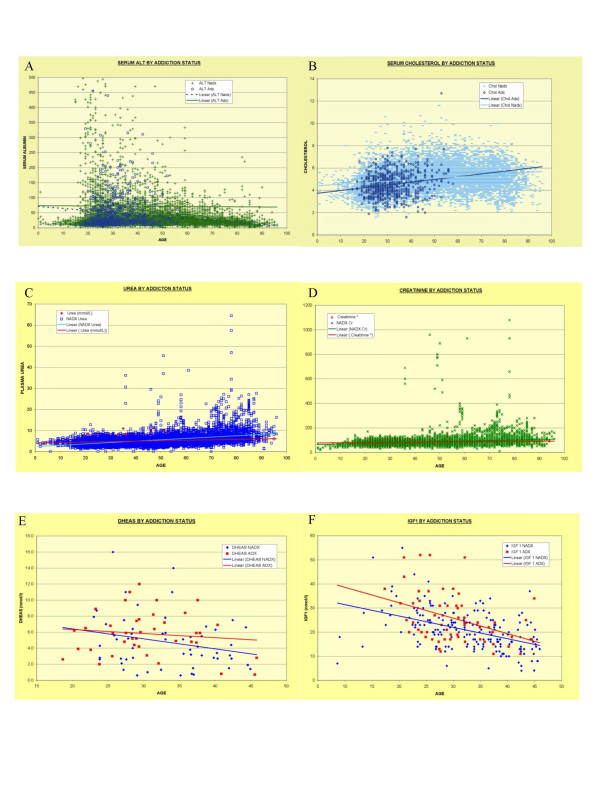
Laboratory Parameters of Addiction, All Ages: Part II – Metabolic. A: Alanine Aminotransferase – ALT; B: Cholesterol; C: Urea; D: Creatinine; E: DHEAS; F: Insulin Like Growth Factor-1

Figures 1 and 2 demonstrates the lifetime trends for the variables of interest, namely CRP, ESR, globulins, albumin, globulin/albumin ratio, total lymphocyte count, ALT, cholesterol, urea, creatinine, dihydroepiandrosterone (DHEAS) and insulin like growth factor-1 (IGF1) together with their lines of best fit. Similar curves may be generated for the period of interest 15–45 years and these are included as Additional File 1. These two figures are best viewed together. The data is quantified in Table 4, and the relative fractions which are elevated either above normal or beyond a certain limit are given in Table 5.

**Table 4:** Clinical laboratory biomarkers in addiction < 45 years of age

	ADDICTS	MEDICAL	P
Age (<45 Years)	30.92 (0.24)	30.74 (0.11)	N.S.
CRP(mg/L)	10.15 (2.11)	7.57 (0.55)	N.S.
ESR	11.34 (1.29)	8.75 (0.35)	0.05
Albumin(g/L)	42.97 (0.13)	42.80 (0.14)	N.S.
Globulins(g/L)	30.33 (0.19)	29.47 (0.05)	<0.0001
Albumin/Globulin Ratio	1.45 (0.01)	1.47 (0.00)	0.01
Globulin/Albumin Ratio	0.72 (0.01)	0.69 (0.00)	0.02
Lymphocytes(x10^9/L)	2.41 (0.03)	2.26 (0.01)	<0.0001
ALT (U/L)	74.26 (7.99)	51.05 (2.69)	<0.01
Cholesterol (mmol/L)	4.48 (0.04)	4.77 (0.14)	<0.0001
Urea (mmol/L)	4.76 (0.04)	4.48 (0.19)	<0.0001
Creatinine (mcmol/L)	81.93 (0.49)	78.91 (0.27)	<0.0001
IGF1 (nmol/l)	25.81 (1.35)	20.79 (0.60)	<0.002
DHEAS (mcmol/l)	5.81 (0.44)	4.89 (1.01)	N.S.

Mean (± S.E.).

**Table 5:** Laboratory parameter elevationsSelected results, patients <45 years

PARAMETER	Addicts†	Medical†	%'s*	OR	CI	P
Age <45 Years	739/798	5834/11295	92.6%/51.7%	11.72	8.90–15.47	<0.0001
Body Mass Index >25	148/297	35/53	33.3%/39.8%	0.75	0.46–1.24	N.S.
CRP > 6 IU/l	81/73	490/811	52.6%/37.7%	1.84	1.30–2.60	<0.0005
ESR>30 mm/hr	13/155	49/1159	7.7%/4.1%	1.98	1.00–3.87	<0.05
Globulins >35. g/dl	90/519	503/4930	14.8%/9.3%	1.70	1.32–2.18	<0.0001
Albumin > 42 g/dl	345/264	3000/2433	56.7%/55.2%	1.06	0.89–1.26	N.S.
Globulin/Albumin Ratio >0.9	90/519	503/4930	14.8%/9.3%	1.69	1.21–2.21	<0.002
Albumin/Globulin Ratio >1.8	57/552	495/4938	9.4%/9.1%	1.03	0.76–1.39	N.S.
Lymphocytes >2.5 × 10(9)/l	224/377	1281/2748	37.3%/31.8%	1.27	1.06–1.53	<0.01
ALT > 90 IU/l	105/505	504/4920	17.2%/9.3%	2.03	1.60–2.57	<0.0001
Cholesterol > 4.2 mmol/l	347/263	3593/1872	56.9%/65.8%	0.67	0.56–0.80	<0.0001
Creatinine > 100 mcmol/l	59/679	272/5159	8.0%/5.0%	1.65	1.22–2.23	<0.001
Urea > 5 mmol/l	227/381	1588/3727	37.3%/29.9%	1.41	1.17–1.67	<0.0002
Urea/Creatinine Ratio >6.5	81/658	288/5140	11.0%/5.3%	2.78	2.11–3.65	<0.0001
Creatinine/Urea >22	134/605	1251/4177	18.1%/23.0%	0.74	0.60–0.91	<0.005
IGF1 >30 nmol/l	19/40	21/162	32.2%/11.5%	3.66	1.70–7.91	<0.0002
DHEAS >6 noml/l	30/40	57/109	42.9%/34.3%	1.54	0.78–2.64	N.S.

† – Cells in these columns represent the numbers greater and less than the defined cut-off point.

* – Relates to Percentages in Addicts Vs. Medical Patients

There is little relative change in the CRP, although it was shown that 52.6% of DA Vs. 37.7% of GM had elevated CRP (OR = 1.84, CI 1.30–2.60; P < 0.0005). The ESR showed an obvious rise in the <45 years comparison confirmed by a higher mean (± S.E.) 11.34 ± 1.29) vs. 8.75 ± 0.35, P = 0.05, with more elevated values (7.7% Vs. 4.1%, OR = 1.98, OR = 1.98, CI 1.00–3.87; P < 0.05). Serum globulins showed an obvious rise with age with a higher mean in DA 30.33 ± 0.19 Vs. 29.47 ± 0.05 with 14.8% Vs. 9.3% elevated (OR = 1.70 CI 1.32–2.18; P < 0.002). The albumin levels were identical in both groups, so the globulin/albumin ratio rose in DA over both the total lifespan and particularly in the <45 years group (14.8% Vs. 9.3% >6.5, OR = 1.69, CI 1.21–2.21; P < 0.002). The lymphocyte count in the GM patients showed the usual decline with age. However, this was absent from the DA group, where the lymphocyte count remained stable throughout life. Hence the mean lymphocyte count was higher in the DA 2.41 ± 0.03 Vs. 2.26 ± 0.01 × 10^9^/l and more DA had a lymphocyte count above 2.5 × 10^9^/l (37.3% Vs. 31.8%, OR = 1.27, CI 1.06–1.53; P < 0.01).

The body mass index was not different between the two groups (Additional File 1, Table 5). ALT is significantly higher in the DA group by all measures. The mean DA cholesterol (4.48 ± 0.04 mmol/l) is less than that in GM (4.77 ± 0.14; P < 0.0001), but as noted the albumin is unchanged between the two groups. The urea and creatinine are both higher in DA, and elevated ratios of urea to creatinine are more common in the DA group 11.0% Vs. 5.3% (OR = 2.78, CI 2.11–3.65; P < 0.0001).

**Figure 3:**
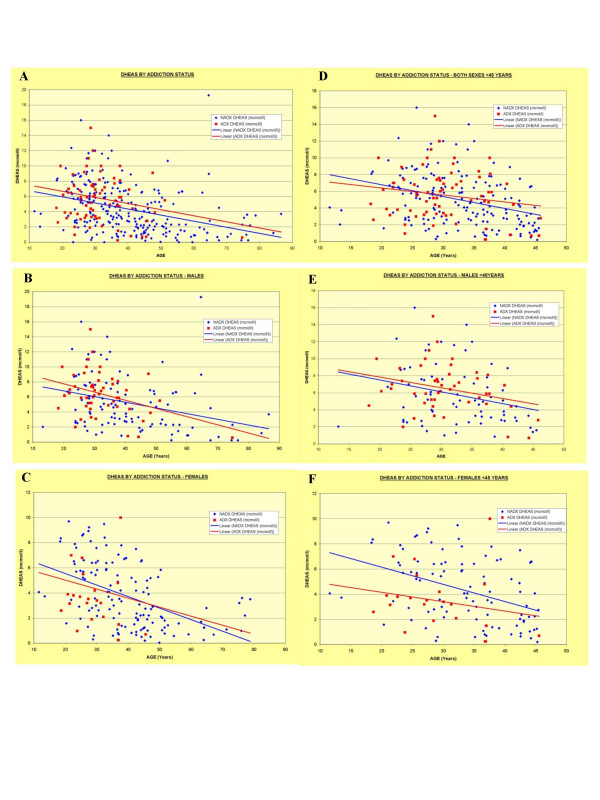
DHEAS by addiction status and age and sex groups. A: All Ages Both Sexes; B: Males of all ages; C: Females of all ages; D: both Sexes <45 Years; E: Males <45 Years; F: Females < 45 Years.

IGF1 was higher in the DA group. The rate of IGF1 above 30 was greater in DA (OR = 3.66, CI 1.70–7.91; P < 0.002). DHEAS levels were not different overall, or in any of the age and sex groups examined. As it is an androgen, with higher levels in males particularly during adult years, a detailed graphical analysis of these data is presented in Figure 3.

## Discussion

These data demonstrate in HIV negative populations that addicted patients have a clinical pathological profile of immune stimulation (higher ESR, CRP, globulins, globulin:albumin ratio, lymphocyte count) in the setting of hepatic (higher ALT and lower cholesterol) and renal (higher urea, creatinine and urea:creatinine ratio) dysfunction. The body mass index and albumin were similar in the two groups suggesting nutritional adequacy in the DA group. The DHEAS showed no overall between group variation, but interesting differences in patients less than 45 years. Interestingly the IGF1 appeared better in the DA group.

The major finding of this study is that as these various parameters have been suggested as important biomarkers of the ageing process, they form together a highly suggestive pattern of findings that the ageing process in addiction might be following a different time trajectory from medical patients. They also suggest that the known immune derangements of addiction may be playing a role in the observed systemic perturbations.

There is a large literature particularly coming from the study of centenarians in Italy which posits that chronic antigenic stress exerts a strain on the immune system, uses up all the naïve T-cells and causes long term CD4 and CD8 memory cells to accumulate [44-52]. In part this is believed to occur via free radical stimulation of P16INK4A dependent apoptosis [53]. It is fascinating to consider that all these circumstances pertain in addiction. In the absence of HIV infection chronic antigenic stimulation in addiction can originate from dental, bronchial and cutaneous foci, from chronic hepatic inflammation and viral shedding, from the use of drugs, and the endobronchial absorption and intravascular injection of particulate substances all of which are directly active on immune cells and the injection of particulate impurities including microbial organisms and antigens. Hence the situation in addiction closely parallels that in ageing, as both demonstrate chronic antigenic overload, immune stimulation, immunosuppression and signs of accelerated ageing. Hence the immune system may not only be a spectator in both the ageing process and in addiction, but a major effector of time dependent decay. Clearly these parallels require more detailed prospective investigation.

It is also interesting to speculate on the interrelationship between the immune stimulation and solid organ dysfunction documented in the pathological study. The liver in particular is known to harbor a substantial body of immune (Kupffer) cells and endothelial cells in its sinusoids, and their interaction with and response to systemic alterations may be more than passive. Both the immune and endothelial components are believed to be important in modern theories of ageing. This is particularly relevant in a context where the liver is receiving long term irritation through alcohol, intravenous injection, drug detoxification or chronic viral infection. Clearly several drug related factors must inform the use of the ALT as an uncomplicated biomarker of ageing in the context of addiction. Albumin is synthesized in the liver but was found to be identical in the two groups. Cholesterol, although not different in DA and GM, was noted to be above 4.2 mmol/l less frequently (Table 5) and to rise more rapidly with age despite a somewhat slower rise in BMI in the DA group (Additional File 1). It is conceivable then that the liver is a primary or secondary driver of other observed systemic changes as has been previously noted [54,55].

The differences observed in the hormonal profiles are also of interest. Considerable research and community attention has surrounded DHEAS as a major adrenal androgen associated with age related changes [56,57]. Its levels may rise with immune stimulation [58,59]. DHEAS protects lymphocytes from apoptosis [60] and also LDL particles from pro-atherogenic oxidation [61]. DHEAS is also elevated by exercise [62] which may be higher in these patients. There was a suggestion that under 45 years DHEAS was higher in males and lower in females than controls (Figure 3) but these did not reach significance. Larger studies would likely have more statistical power to detect such alterations.

Similarly IGF1, the mammalian homologue of the invertebrate DAF-16, is one of the main hormones implicated in insulin – insulin-like-growth-factor signalling (IIS) pathway in the biology of ageing [63]. It was not possible to study insulin or C-protein levels as we did not have access to the fasting state in this group of outpatients. IGF1 is a key component of the longevity regulation system conserved from yeast to mammals, and has recently been implicated mechanistically in the pathogenesis of various experimental neurodegenerative disorders [64,65]. Higher levels of IGF1 in DA in this study are therefore conceptually provocative.

Whilst this study has the virtue of relatively large numbers in a single centre, it is not possible to delineate causal pathways from a purely empirical study of this nature. Clearly further experimental and clinical work would be required to formally evaluate this hypothesis. Furthermore it is unclear whether the pharmacology of addiction itself, or some other related lifestyle factor such as poverty, homelessness, imprisonment, poor nutrition or overdose is largely responsible for the observed effects. Nevertheless the apparent uniformity of results across diverse body systems is intriguing. A further limitation of this study was the use of medical patients as controls. It would be an advantage if future funded clinical studies had detailed information of patients' drug use available for all samples to allow multivariate association studies to be performed, and formal assignment of patients' addiction status.

This work would appear to invite further clinical and laboratory based investigations of the dynamic interaction between addiction and ageing, with the hope that improved understanding of these related disorders can improve our clinical management of ageing and addicted populations, and our understanding of the role of immune suppression and hyperstimulation in the ageing process.

## Supplementary Material

Additional file 1 Supplementary Figure 1. Clinical Pathological Indices <45 Years. A: C-reactive protein – CRP. B: Erythrocyte Sedimentation Rate – ESR. C: Globulins. D: Albumin. E: Albumin/Globulin Ratio. F: Globulin/Albumin Ratio. G: Lymphocyte Count. H: Alanine Aminotransferase – ALT. I: Cholesterol. J: Creatinine. K: Urea. L: Creatinine/Urea Ratio. M: Body Mass Index – BMI. N: Body Mass Index, All Ages. Click here for file
