# Supplementary figures and images for: Evidence of accelerated ageing in clinical drug addiction from immune, hepatic and metabolic biomarkers
Source: Immun Ageing. 2007 Sep 24;4:6. doi: 10.1186/1742-4933-4-6 (PMC2041948; doi:10.1186/1742-4933-4-6)

## Slide 1
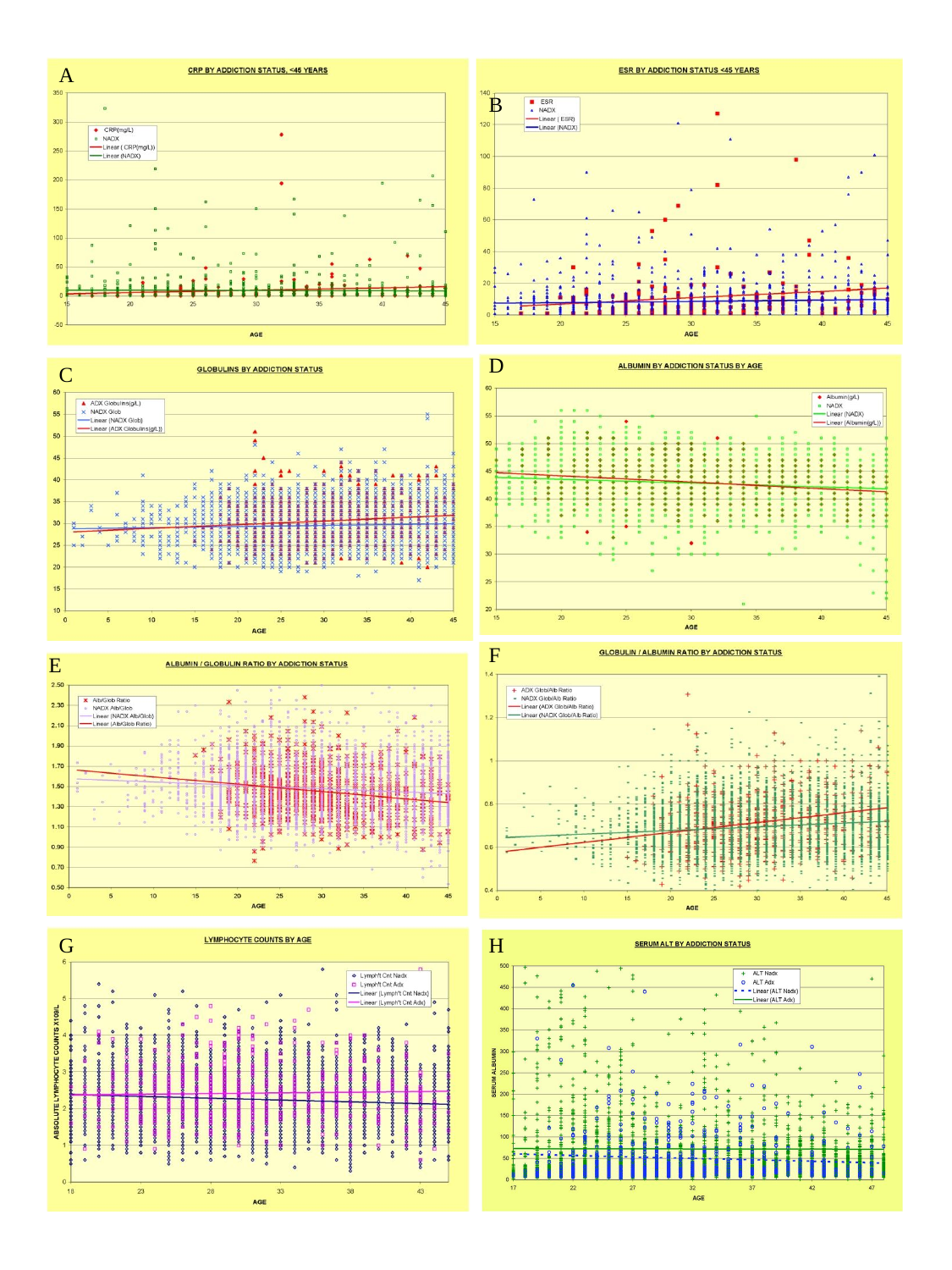

A
B
D
C
F
E
G
H

## Slide 2
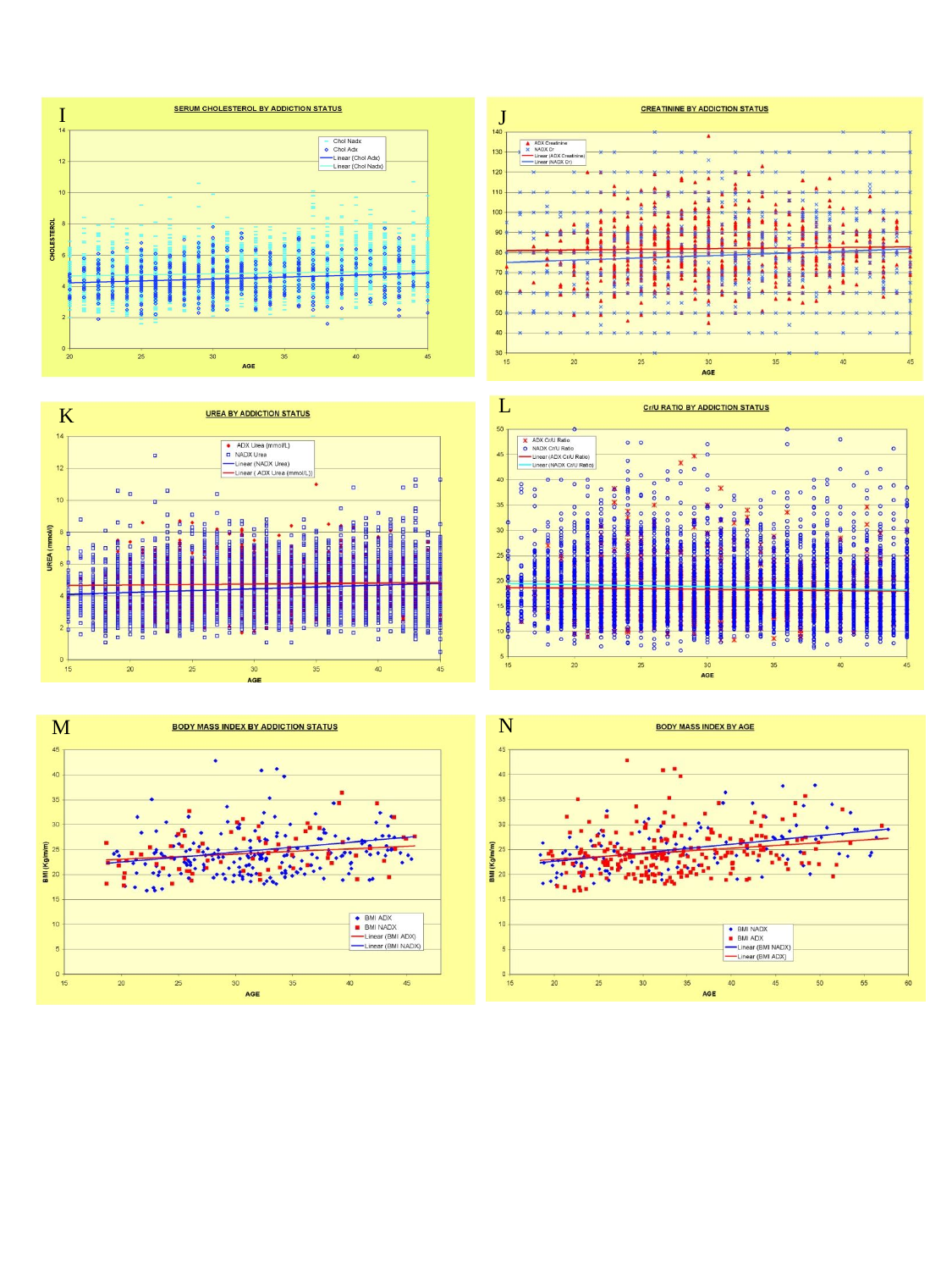

I
J
L
K
N
M

Supplement: Additional file 1 — Supplementary Figure 1. Clinical Pathological Indices <45 Years. A: C-reactive protein – CRP. B: Erythrocyte Sedimentation Rate – ESR. C: Globulins. D: Albumin. E: Albumin/Globulin Ratio. F: Globulin/Albumin Ratio. G: Lymphocyte Count. H: Alanine Aminotransferase – ALT. I: Cholesterol. J: Creatinine. K: Urea. L: Creatinine/Urea Ratio. M: Body Mass Index – BMI. N: Body Mass Index, All Ages. [file 1742-4933-4-6-S1.ppt]
